# A stochastic mathematical model of avascular tumor growth patterns and its treatment by means of noises

**DOI:** 10.22088/cjim.8.4.258

**Published:** 2017

**Authors:** Yasaman Moghadamnia, Dariush Moslemi

**Affiliations:** 1 Alzahra University, Tehran, Iran.; 2 Cancer Research Center, Health Research Institute, Babol University of Medical Sciences, Babol, Iran.

**Keywords:** Cancer modeling, avascular tumor growth, White noise, Stochastic modeling, Cancer treatment.

## Abstract

Due to the rate increase in cancer incidence, many researchers in different fields have been conducting studies on cancer-related phenomena. Most studies are conducted to focus on cellular and molecular aspects of cancerous diseases and treatment strategies. Physicists have been using mathematical modeling and simulation to explain the growth pattern of tumors. Although most published studies in this area still have not gained the needed maturity for “treating cancer”, research has helped with the statistical laws of growth, predation and proliferation of cancer cells. In this review, a brief explanation of mathematical models for tumor growth is presented, followed by a discussion on treatment simulations, introducing white noise as one of the clinical remedies in the original model.

## Introduction

**Figure 1:**
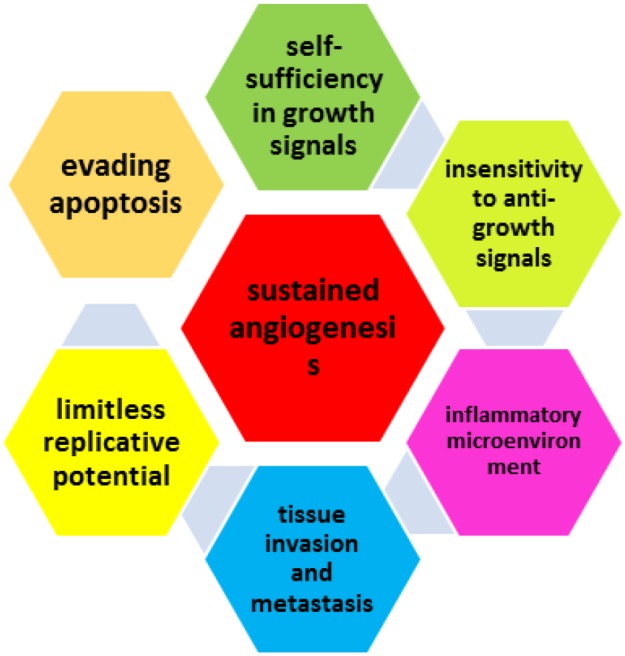
The seven hallmarks of cancer as defined by

**C**ancer is the second leading cause of death in the world after cardiovascular diseases (1). Malignant tumors mainly originate from nonlethal genetic damage (or mutations) that most probably happen due to environmental defecting factors and/or spontaneous mutations inside the cells. Generally four types of cell growth regulatory genes are subjected to damage in the process of carcinogenesis; the growth-promoting proto-oncogenes, the growth-inhibiting tumor suppressor genes, genes that regulate programmed cell death (apoptosis), and genes involved in DNA repair (2). A cancerous tumor generated as a result of the mentioned genetic mutations, if not subjected to external disturbances or deprived from food and oxygen, will continue growing and will eventually gain properties that according to (3) the seven hallmarks of cancer. Thus a tumor is specifically malignant and can potentially cause death if it contains the properties shown in figure 1. From a statistical physics point of view, cancer is a complex system that behaves stochastically at both small and large scales. The components of a complex system are said to be simple entities, sometimes behaving completely stochastic, yet somehow staying connected with each other despite the lack of a central controlling system (4). This could be observed in a tumor that initially created from a single precursor cell and is now expanding due to its cancerous components growing and proliferating. In addition to such harmony in a complex system, a more interesting feature is the building blocks of the system’s ability to evolve into new pathways that are beneficial to the system as a whole. Through past three decades, much effort has been made to present a mathematical model of the behavior of cancer as a complex system. According to (5), models worth noting for biologists, biochemists and medical doctors are those that can explain the phenomena occurring during the evolution of tumors using three natural viewpoints: cancer phenomenon on three levels: the sub-cellular, the cellular and the tissue level (5). 

Many various and vast studies have been conducted on modeling different aspects of cancerous systems, in an attempt to clarify their functionalities more accurately. According to (6), these include “initiation and promotion”, and “a single cell origin of cancer” models for explaining tumor origination, “the single-stage theory of cancer” and “the multi-cell transformation theory” models that were mainly proposed for cancer development, etc. .From the viewpoint of reductionism, every aspect of a cancerous system functions differently from the rest, and thus, demands different scientific theories to be well studied, for instance, a study of angiogenesis and its effects could be done using tools of hydrodynamics, whereas suggested tumor growth mechanisms are understandable more easily through the view of non-equilibrium statistical mechanics (7-10). 

**Figure 2:**
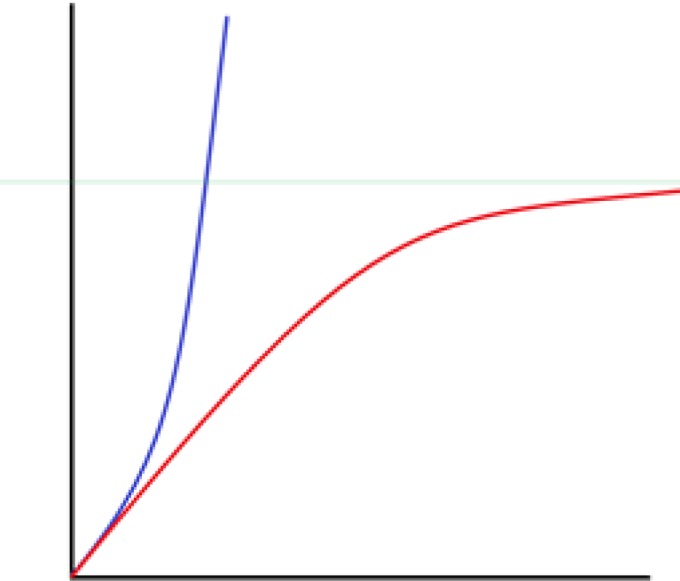
The Gompertzian growth Vs. The exponential growth

It seems trivial that learning more about the mechanism of tumor growth can help develop more efficient treatment methods (11). Models that were primarily suggested for the growth pattern of a simple avascular tumor were of the Gompertz growth model category, also known as the logistic growth formula, which was inspired by population growth in insect colonies (5). This model states that initially the population in a given tumor grows exponentially and rapidly with time, however, it eventually reaches a state of saturation (Fig.2). Although this model is somewhat simplified and fails to explain the tumor’s behavior after angiogenesis, it has been proven to be more or less accurate and helpful, especially during the first stages of cancer development (7, 8, 12-16). In this review, the Gompertz model was studied and compared with some well-known growth models. Afterwards, a suggested mathematical method of adding white noises to the growth model was explained as a possible way of simulating treatment in the main growth model. When a single damaged cell, namely the precursor cell, starts to proliferate so as to create a tumor, it resembles a mathematical geometric progression, meaning a primary cell will be doubled and each of its daughter cells are doubled and so on (2). Mathematically thinking, such a system that is growing exponentially has the capacity of growing to infinity, outgrowing the capacity of its holder, hence reaching population explosion. Equation 1-1 may be used to interpret that characteristic (5): N(t)= N_0_e^kt^

Where $N\left(t\right)$ is the number of cells present in the tumor at time t, $N_{0}$the number of cells initially present in the tumor (at time t=0) and k is the net rate at which the tumor grows. Nenetheless, this is not the real life context, at least not with populating systems such as a cancerous tumor. The reason lies within two important factors that were initially omitted from the progression model. The first one is to account for the number of “deaths” within the system, a factor that can negatively affect the population. As for cancerous cells, it is a known fact that they evade apoptosis and are in theory, undying, yet they still could be eliminated through the process of growth. The larger the tumor becomes, the harder it would be for the inner cells to reach food and oxygen, which will eventually lead to their death, a phenomenon that could be seen as necrosis in the tumor tissue (2). There is also a second factor that will limit the tumor’s swelling up to infinity, which is the “carrying capacity of the environment”. 

The carrying capacity is defined as the environment’s ability to host the growing system, e.g. in the case of cancer, the tumor expansion is limited by the surrounding tissues’ finite space to spare, accessibility to food and oxygen, etc.. Adding these two restraining terms to the original growth formula (the unlimited exponential growth) will result in a growing pattern that eventually reaches a saturation point. This formula was primarily suggested by B. Gompertz in 1825 and then later developed into a growth model for avascular tumors (10^6^ cells and more) by Laird in 1964 (6). The simplified version of this model (the logistic growth model) is as follows:

1-2 $$\left(\frac{\mathrm{dN}}{\mathrm{dt}}=\mathrm{kN}(1-\frac{N}{\theta }\right)$$

In 1-2, **K** is the net rate of cells’ proliferation, **N** the number of cells, and **ϴ** the carrying capacity of the population. This equation is mainly stating the different factors that affect the number of cells in the whole population respective to time. The term is one that is crucial for designing a proper treatment method. More specifically, if a treatment term is added to this formula, it needs to reduce the number of cancerous cells through time, and possibly stop the growth of the tumor, a concept that corresponds to. dN/dt=0

*Growth Pattern during the latency phase:* ***It has been reported that it might take up to a decade for a malignant tumor to be detected (*** 1 ***). Growth models such as the Gompertz equation explain tumor expansion in an observable phase, which naturally follows the initial latent phase. Investigations as such as (*** 16 ***) have been done to determine the growth mechanism during the latent phase, mostly using in vivo implantation of tumor cells and measuring the size of the tumor and its growth rate. In general, many such studies provide evidence supporting the interpretation that was made earlier on the growth’s exponential pattern through the first stages, which is equivalent to the latency period (*** 6 ***). As a result, there seems to be a connection between when the tumor is expanding exponentially and it being latent at the time, and the fact that the tumor will grow logistically after being restricted, which is when it could be properly detected.***

**Gompertz model as a stochastic process: **Following the mentioned property of complex systems of its components being able to evolve in ways to preserve the system, Speer et al. (17) introduced a new stochastic component to the Gompertz model. Their research proposed that saturation limit quantity for the logistic growth pattern is in fact a property of each cell that could be further changed and developed via consequent mutations (6). The ability of cells to mutate to contribute to the tumor’s growth process is discussed in such models as a stochastic process. A typical stochastic process is one where a system’ evolution in time is indicated by a variable that changes randomly with time (18).

**Adding treatment terms to the main model: white noise:**

In an attempt to model the effects of treatment on tumor growth, many studies (7, 10, 12) have used stochastic noises as environmental disruptions in their growth model. This procedure allows the simulating process to undergo changes (in an attempt to overcome a cancerous tumor invading healthy tissues) without actually affecting the original growth model. The word “noise” in such studies is referred to any (considerably small) effects induced by environmental disturbances and does not necessarily refer to a real sound or light wave-like noise (15). 

In the case of randomly-behaved systems such as cancer, the most suitable noise to take into account is the white noise. According to (2), “in signal processing, white noise is a random signal having equal intensity at different frequencies, giving it a constant power spectral density”. More specifically, all the possible frequencies representing a different probable behavior of the studied noise are equally probable in the white noise spectrum. This property of the white noise is crucial when taking treatment procedures into account in tumor growth models. In studies like (7, 10, 12, 15), white noises have been represented to logistic or Gompertz growth models as treatments. Although these noises are far too simplified to represent the real clinical procedures performed by physicians nowadays, they can be of acceptable accuracy when applied to simple avascular tumors (the early stages). The white noise, in addition to its randomness property, is said to have a “memoryless” nature, meaning that the random perturbation it applies to the growth process does not "remember" what the effects of the noise was at any other time (19). In the case of real world treatments, the tissue that undergoes a white noise treatment (possibly chemotherapy or radiotherapy) is not memoryless, rather, it remembers the process of receiving a special amount of dosage each time. This is also the reason for gaps between chemotherapy sessions, so as to allow the surrounding tissues to regain their state of strength before experiencing the next therapy session (11). 

The white noises that are added to the original logistic model (equation 1-2) could be of different types. The noises could either be additive or multiplicative in mathematical words (6). The multiplicative noise is simply one that is multiplied by a special term in the model formula, for instance a term related to the oxygen received by cancerous cells. Multiplying this term by a white noise, simply enhances its effectiveness and therefore its contribution to the total growth process of the tumor.

The additive noise is added to the entire system, not only as one specific term. Such added noises are the main representatives for therapeutic procedures in cancer treatment since they are delivered to the entire tumor tissue and most probably the healthy surrounding tissues as well. 

*Treatment in logistic tumor growth model: the role of two white noises:* ***The reported results from investigating the effects of noise treatment on growth models such as the Gompertz and logistic formulas, though subtle, have been in agreement with the accepted cancer therapy theories and techniques (*** 7 ***-*** 10 ***,*** 14 ***,*** 15 ***). For example, (*** 15 ***) has investigated the effects of two white noises on a logistic growth model. The first noise was multiplied by a positive factor that contributed to the tumor’s growth and wellbeing, while the second one is an additive term that appeared as negative to limit the tumor’s growth and eventually lead to its death.***

**Figure 3:**
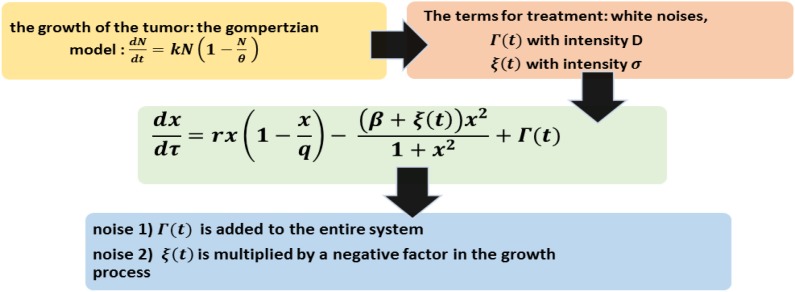
Treatment in logistic tumor growth model

***The model is described schematically in*** figure 3 ***.***

**The reported results to this study were as follows:**

- For a small value of β (already existing negative factor), the effects of the disturbances on tumor growth are unfavorable for tumor growth.
- A large value of β (already existing negative factor) is favorable for tumor growth (in case of noise).
- For a moderate value of β, the effect is determined by the fluctuation in the relative strength of the two noises:
- A) If σ (noise number one’s strength) is small, the increase of D is unfavorable for tumor growth. In contrast, it is favorable for tumor growth if σ is large.
- B) If D (noise number two’s strength) is small, increasing σ is favorable for the tumor growth, in contrast, decreasing σ is unfavorable for tumor growth.

**The interpretation for each result is as follows:**

1. The main factor that determines the effects of any incoming noise is the negative factor that already existed in the original logistic growth model, namely the limiting factors of cell deaths and the environment’s capacity for a tumor, let us call that factor B.

2) If B is a small factor, the effects of noises will be more in favor of the patient, namely, unfavorable for the tumor. It can be inferred that if B is small, it must mean the negative factors to the growth are negligible and the tumor is growing increasingly. 

The tumor, having a noticeable negative tissue activity, would be easier to detect and as a consequence easier to remove, using the noises (represented as treatment).

1. If B is a large factor, the negative action towards the tumor has been growing increasingly before the noises were added to the system, meaning the immune system is more alert and so will react to any incoming noises more acutely. This means that in this case, the treatment will have a lesser effect and might even contribute to tumor growth by eliminating healthy tissues.
2. In the case of a moderate B, the outcome of the tumor growth will depend highly on the relative intensity between the two noises.

- If the intensity of the multiplicative noise (let us call it M) is a small number, increasing the intensity of the additive noise (let us call it A) will help with the treatment and termination of the tumor.

It would be the ideal treatment. More precisely, the negative factor in the original model (before adding the noises) is small, and so the immune system is not totally activated, and the tumor could be destroyed using the additive noise.

- If the A is small (the treatment term), increasing the other noise’s strength (the M which contributes to the negative factors) will be unfavorable for the patient and thus block the real treatment process.

## Discussion

Growth models that have been suggested for cancerous tumors have been developing through vast parts of mathematics and physics. 

Nevertheless, the most fundamental model given for a tumor’s growth pattern at the most rudimentary stages of its life is the Gompertz model that indicates a tumor reaching a saturation point after having grown exponentially with time. The accuracy of this model has been proven to a great extent through various studies. 

The main expectation of physicians from a model on cancer behavior is its way of contributing to treatments. Since cancer is considered to be a stochastic complex system, the suggested terms to be added to its original growth model as treatment were most fundamentally randomly-natured noises. Studies (6-10, 12, 14, 15) have conducted such simulations by adding white noises to the original system and reporting the effects of noises on terminating a non-specific avascular tumor.

Through the various results that were given by such studies, the most important one seems to be the dominant role of the term B, or the existing negative factor in a growth model, reported at most studies investigating the treatment effects on the logistic growth model (6-10, 12, 14, 15). This factor is mainly the reason a tumor reaches a stop point after initially growing rapidly. There seems to be direct correlations between this factor and the level of sensitivity shown by the immune system around the affected tissue (2). It then seems highly logical to consider limiting the B factor before adding any treating noises (could be any therapeutic procedure in cancer treatment) to the cancerous tumor, otherwise, the results might have the perfect opposite outcome that the expected one. These models may appear helpful to oncologists and cancer diagnosticians, in treating avascular tumors (in case of detection at the early stages, before developing angiogenesis). 
